# Facilitators and barriers to patient-centred goal-setting in rehabilitation: A scoping review

**DOI:** 10.1177/02692155221121006

**Published:** 2022-08-25

**Authors:** L. Crawford, J. Maxwell, H. Colquhoun, S. Kingsnorth, D. Fehlings, S. Zarshenas, S. McFarland, Nora Fayed

**Affiliations:** 1 School of Rehabilitation Therapy, Queen's University, Toronto, Canada; 2 Holland Bloorview Kids Rehabilitation Hospital, Toronto, Canada; 3 Bloorview Research Institute, Toronto, Canada; 4 Occupational Science and Occupational Therapy, University of Toronto, Toronto, Canada

**Keywords:** Goal-setting, behaviour change, scoping review, barriers and facilitators, implementation science

## Abstract

**Objective:**

Identify, map, and synthesize existing reviews, to extract and analyse the most prominent barriers and facilitators to applying patient-centred goal-setting practice in rehabilitation using the Capability, Opportunity Motivation Behaviour (COM-B) model.

**Design:**

Scoping review.

**Data source:**

A primary search was conducted in MEDLINE, CINAHL, EMBASE, PsychInfo, and Cochrane. Citation chaining was employed.

**Review methods:**

All types of review (systematic, scoping, and narrative) studies published up to June 14, 2022 that included physical and neurological rehabilitation, patient-centeredness, and goal-setting were reviewed. Studies were scrutinized for relevance, quality was not assessed. The most prominent barriers and facilitators were synthesized using thematic content analysis and mapped onto the COM-B model.

**Results:**

Twenty-six review studies covering a range of conditions and settings, acute to community were included. Barrier and facilitators were identified at patient, provider, and organizational level. Barrier themes include provider's existing beliefs about goal-setting, lack of skills, and integration into clinical routines. Patient barriers related to capacity and opportunity to participate. Organizational barriers include lack of clinical guidelines, patient preparation, insufficient provider time, and high productivity expectations. Facilitators included goal-setting guidelines, training and education of providers and patients, revised clinical routines, performance monitoring, adequate time, and resources.

**Conclusion:**

Healthcare providers should be the primary target of intervention. A provider's motivation to change current practice is the most prominent barrier, followed closely by capacity and opportunity. Patients require information, training, and structured engagement opportunities. Organizations play a key role in creating the optimal environmental conditions to enable patient-centred goal-setting.

## Introduction

Collaborative goal-setting is a fundamental aspect of patient-centred rehabilitation, but healthcare providers (herein referred to as HCPs) have been slow to adopt it in practice.^1–11^ Providers are often motivated to lead goal-setting to achieve expected professional and/or organizational outcomes.^1–4^ In contrast, patient-centred goal-setting involves clients in a process of goal identification and agreement, respecting client values and preferences, and resulting in personally meaningful outcomes that are measured and reported.^6^ Patient-centred goal-setting helps providers and patients focus their behaviour on meaningful outcomes that improve the patient's quality of life.^1–4,7^ Evidence shows engaging patients in goal-setting improves their confidence, motivation, and satisfaction with rehabilitation.^1–4,7^ Providers benefit from the enhanced patient participation and improved team functioning resulting from a collaborative approach to goal-setting, which contributes to improved client outcomes.^1–4,6,7^ This evidence is based on two systematic reviews about the effectiveness of patient-centred goal-setting in adult stroke rehabilitation.^1,2^ In a Cochrane review,^4^ the authors found that there is reasonable support for the effectiveness of a structured approach to goal-setting and its benefits, but the results were inconclusive due to the low methodological quality and study heterogeneity.^1,2,7^ Also, the disparate language and terminology in these reviews makes it difficult to conclusively assess which barriers and facilitators are most frequently identified, and the knowledge gaps that need to be further studied.^7^

There are several international systematic reviews, particularly in neurological rehabilitation, about barriers to patient-centred goal-setting, which limit the adoption of this practice despite the need to do so.^1–6,9–11^ These barriers to patient-centred goal-setting include patient preparedness^1–4,6,7,11,12^; differences between provider and patient perspectives^1–4,8–15^; provider skills^1,3,4,6,7,9,10,12,13,15–17^; and various organizational barriers, such as time, workload pressures, and lack of approved goal-setting frameworks.^1–4,6,7,10,15^ Thus, the evidence indicates the uptake and use of patient-centred goal-setting in routine practice is challenging despite its benefits. Therefore, the known barriers and facilitators need to be studied using a theoretical behaviour change model, but no such summation has been found in the literature. This omission makes it difficult to understand which behaviours need to be targeted by behaviour change interventions to enable goal-setting, and at which level (patient, provider, organizational). A scoping review allows for the identification of the number of pertinent reviews in the literature for inclusion in the analysis.^18,19^

The Capability, Opportunity, Motivation Behaviour Model is a comprehensive, systematic, and commonly used framework for understanding goal-setting behaviours and identifying which behaviours to target for intervention design.^20^ The objective of this scoping review is to map and describe the existing evidence about the barriers and facilitators to patient-centred goal-setting in rehabilitation as aligned with the Capability, Opportunity, Motivation Behaviour Model, for the purpose of informing targeted behaviour change interventions.

## Methods

Standard scoping review methods^18,19^ were modified to only include review studies due to the breadth of reviews available about patient-centred goal-setting. This approach decreases duplication and allows for synthesis of existing information. The methodological quality of the review studies was not evaluated.^19^ Reporting was completed in accordance with the PRISMA extension for scoping reviews^21^ (Supplementary File 1). The review protocol was not registered.

The primary research question is: What are the facilitators and barriers to applying patient-centred goal-setting in rehabilitation at the patient, provider, and organizational level?

Two authors (LC and SK) searched MEDLINE, CINAHL, EMBASE, PsycInfo and the Cochrane Database of Systematic Reviews databases from inception to 14 June 2022. In addition, reference lists of relevant articles were hand searched and screened to identify additional articles. Grey literature was excluded.

The initial search strategy was developed for MEDLINE and consisted of using the keywords Goals OR Participation OR Keyterms (Goal setting OR plans OR negotiat* OR discuss* OR propos* OR prescribe* OR develop* OR formulate* OR establish* OR identif* OR plan* OR coping plan* OR shared decision mak*) AND [Mesh Headings] (Stroke rehabilitation OR Rehabilitation / organization & administration OR keyterms [kf,tw] rehabilitation). Filter applied for systematic reviews, reviews, qualitative studies as publication types. Additional keywords were derived from relevant titles and abstracts. The search strategy was refined through multiple preliminary iterations with the assistance of the Holland Bloorview Kids Rehabilitation Hospital librarian. The MEDLINE search strategy was adapted to search the remaining databases. The search strategy for each database is shown in Supplementary File 2. All searches were conducted by the first author.

All articles were screened by four reviewers (LC, SM, HC, and SZ) and additional reviews were identified through citation chaining. The authors discussed conflicts to achieve consensus on article inclusion.

**Study inclusion criteria:**

- Any peer-reviewed published review study
- Review studies containing barriers and facilitators to patient-centred goal-setting in rehabilitation using any type of goal-setting intervention
- All client ages, with any physical and/or neurological disability who access rehabilitation
- Any health professionals delivering rehabilitation
- All rehabilitation settings (e.g., acute, outpatient, community, school)
- Only publications written in English as translation facilities were unavailable.

Studies conducted in mental health and addictions rehabilitation were excluded because the goals and interventions are significantly different from physical and neurological rehabilitation.

Standard data was extracted by one reviewer from each study: author, year, country of first author, review type, participants, type and number of primary studies, study type and aim(s), setting, and barriers and facilitators to goal-setting.^18,19^ Structured content analysis was used to identify the barriers and facilitators by level (patient, provider, and organization) in each paper a second reviewer checked the data.

Utilizing the results of the content analysis, two reviewers independently grouped the codes and organized them into meaningful sub-categories using the Capability, Opportunity, Motivation Behaviour Model (Table 1).^20^ These sub-categories were then collapsed into major categories and aligned to the patient, provider, and organizational level. The Capability, Opportunity, Motivation Behaviour Model sub-components are not clearly defined so the Theoretical Domains Framework was used to clarify and more comprehensively define the Capability, Opportunity, Motivation Behaviour Model sub-components for the purpose of coding, organizing, and developing major facilitator and barrier categories.^22^ Any discrepancies or themes not easily coded in the content analysis underwent constant comparative analysis until there was interrater agreement. A frequency count was conducted to identify the most commonly reported barriers and facilitators.

**Table 1.** Capability, Opportunity, Motivation Behaviour model (COM-B) sub-component definitions.^20^

COM-B component	Sub-component	Theoretical Domains Framework Domains
**Capability**	Psychological	Knowledge
		Cognitive and interpersonal skills
		Memory, attention, and decision processes
		Behavioural regulation
	Physical	Skills
**Opportunity**	Social	Social influences
	Physical	Environmental context and resources
**Motivation**	Reflective	Social/professional role and identity
		Beliefs about capabilities
		Optimism
		Beliefs about consequences
		Intentions
		Goals
	Automatic	Reinforcement
		Emotion

## Results

In the initial search we identified 1691 articles, which resulted in 44 included studies. After full-text review 26 papers are included in the review. The details of the search results are in a PRISMA diagram in Supplementary File 3.

Of the 26 papers, over half are systematic reviews, the rest were literature, integrative, scoping, or rapid reviews. The reviews were conducted in eight countries: the United Kingdom (eight reviews),^1–3,10,12,15,23,24^ Canada (five reviews),^8,16,17,25,26^ New Zealand (five reviews),^5–7,27,28^ Australia (three reviews),^11,14,29^ the Netherlands (two reviews),^30,31^ Denmark (one review),^9^ Japan (one review),^13^ and the United States (one review).^4^ All the studies were published between 2004 and 2021. Nine papers were about adult stroke and acquired brain injury populations,^1–4,10–14^ one in spinal cord injury,^9^ and six in general rehabilitation populations,^5–7,15,29,30^ four reviews in paediatric rehabilitation,^8,16,25,26^ and six with unspecified populations.^17,23,24,27,28,31^ There were approximately 212 quantitative primary studies,^1,2,5–8,12–17,23,24,26,29,30^ 196 qualitative primary studies,^1–5,8–17,26,29,31^ and 13 mixed methods primary studies^1,8,11,15–17^ included. These numbers are approximate as not all the reviews explicitly identified the number and type of primary study included. Providers and patients were the participants of interest in most of the studies, primarily physical therapists, occupational therapists, speech language pathologists, as well as some physician and nurse participants.^1,3,4,6–9,12–16,23,24,26,30^ Two studies only included patients and one focussed on patient outcomes.^5,9,10^ One study was on the caregiver (family member or paid caregiver) perspective.^29^

Authors of only three reviews had the primary goal of identifying barriers and facilitators to patient-centred goal-setting in practice, all of which were in adult stroke rehabilitation.^1–3^ Nineteen papers were about the experience of goal-setting,^9,10^ approaches to^4,8,11,12,14,15,29^ and effectiveness of goal-setting,^5–7,30^ including six papers on the use of goal-setting instruments^13,16,17,23,26,31^; barriers and facilitators were secondary aims in these studies. A third group of four papers were about the application of theory to patient-centred goal-setting.^24,25,27,28^ Identified barriers and facilitators in these papers were primarily about the lack of, or application of theory to goal-setting in rehabilitation. A summary of the included studies is in Supplementary File 4.

The structured content analysis of the facilitators and barriers extracted from the 26 papers resulted in 191 codes, from which 27 inductive categories were composed, 10 at the patient level, 11 at the provider level, and 6 at the organizational level. There were a significantly higher number of barriers than facilitators^23^ to delivering patient-centred goal-setting identified. These results are presented in detail below.

### Patient level barriers

#### Capability – Physical and psychological

From a patient perspective, individuals identified a lack of knowledge about their condition, prognosis, and rehabilitation process as a barrier to readiness and participation in goal-setting.^1,3–5,8,13,15^ This lack of knowledge appears to support the provider as the “expert” and may promote prescriptive goal-setting and a passive patient role.^1–7,9–11,13–17,24^ Also, the stage of rehabilitation influenced the patient role, with many patients in the early phase of recovery requiring directed, impairment-based goals; this was a predominant theme in the stroke research as patients adapt to new and evolving capabilities.^1–4,10,11^

#### Opportunity – Physical and social

Patients are not considered part of the multidisciplinary team. Poor patient integration can lead to too many discipline-specific, and less personally meaningful goals.^4,5,7,15–17,27,30,31^ Patients also reported limited time and access to providers, resulting in patients and providers prioritizing therapy over goal-setting.^1–3,11,29^

#### Motivation – Reflective

There were differences between provider's perspectives for goal-setting and that of the patients.^1–3,5–9,11,14,16,17^ Patients tend to articulate broader, long-term independence goals,^1–3,8–11^ and the real world.^3,9–12^ HCPs prefer specific, short-term, and impairment-based goals that support the routine clinical workflows and organizational productivity targets.^1–4,6,7,9–11,16,17,24^

#### Motivation – Automatic

Patients do not feel that the environment is safe, and they are afraid to share hopes and challenges during goal-setting.^3,4,7,13,27–29,31^

### HCP Level Barriers

#### Capability – Physical and psychological

There is no agreement on a sector-accepted goal-setting approach, applicable theory, or standardized tool(s).^5,6,8,13,14,16,23^ The lack of standardization makes provider education and training difficult and leads to inconsistent practice.^1–3,5–7,9–17,26,29,31^ Without specific direction, providers continue to rely on interventions specific to their discipline.

#### Opportunity – Physical

The lack of time, unsupportive clinical documentation, and overuse of technical language were identified as barriers to collaborative goal-setting.^1–4,6,8,10,11,14,26,29^ Many authors identified patient goals are not used to enhance interprofessional team functioning, such as coordination and communication.^1,3,8,11,12,16,23,31^

#### Opportunity – Social

The review verified many clinical team cultures exclude patients from authentic participation, impacting engagement and threatening development of effective therapeutic relationships.^4,5,7,15–17,27,30,31^

#### Motivation – Reflective

Providers” attitudes and beliefs about patient-centred goal-setting were the most frequently identified barrier. Providers believe they are already providing patient-centred goal-setting.^1–3,10,12,14,15,31^ Many assume it is their role to be the expert and patients want them to be directive.^1–5,7–12,14–16^ Providers feel patients are incapable of participating in goal-setting because of a lack of knowledge about their condition, goal-setting and rehabilitation processes.^1–4,8–10,12,14,16,17,26,29–31^

#### Motivation – Automatic

Providers did not feel confident in conducting patient-centred goal-setting due to lack of training and understanding of application in practice.^1,3,6–10,12–17,27–31^

### Organizational level barriers

#### Capability – Physical and psychological

Most authors identified the lack of a standardized goal-setting approach, which includes tools,^5,7,12,17,23,29,31^ policies and integration of theory to support practice change, and goal-setting skill development for providers and preparation for patients.^1,4,6–8,12–15,23–26,29^

#### Opportunity – Physical and social

Researchers highlighted numerous barriers, including lack of time^1–3,8,10–12,14,15,17,24,29,31^ for providers due to high workload targets and resource constraints,^1,3,4,6–8,15,24,27,28^ and inadequate clinical documentation.^3,6–8,12,16,29^ A social opportunity barrier is noted in the lack of understanding of the impact of patients’ culture on goal-setting.^2^

#### Motivation – Reflective

Without a standard goal-setting approach, measurement, and recognition of provider performance is not feasible.^3^

#### Motivation – Automatic

The impact of patients’ culture and diversity regarding goal-setting is not studied or understood.^2^

### Facilitators

#### Capability – Physical

Many authors identified providers and patients require a standardized, evidence-based approach to goal-setting that can be tailored to individual preferences.^4,5,12,13,16,17,23,24,26^ Providers and patients would need to be educated to deliver and be involved in this approach.^1–6,10,11,13–15,17,27,29,30^ Providers require enhanced communication and goal-setting facilitation skills to consistently enable individual tailoring.^1–4,7–11,14,15,24,29^

#### Capability – Psychological

Authors identified patients require education on their condition, goal-setting, and rehabilitation processes to develop goal-setting skills and be prepared to participate.^1–4,9–11,14,25–28,31^ The patient role and expectations should be clear but may change through the various stages of recovery.^1,4,6,8,9,13,15,16,25^

The providers need to be educated on the importance of goal-setting, effective use of behaviour change and theory in practice to facilitate patient motivation, engagement, and improved outcomes.^1,3,4,6–8,10,11,14,15,25,27,28^

#### Opportunity – Physical

Adequate time is required for patients and providers to allow for authentic engagement in collaborative, tailored goal-setting.^1,3–8,15,24,27,29^ Routine clinical workflows require redesign to allow for this, and productivity tracking measures would have to incorporate time for goal-setting, signalling it is a valued practice.^1,3,6–8,15,24,27,29^ Appropriate clinical documentation can be an effective tool to enable the practice.^1,6,12,15,16,29,31^

#### Opportunity – Social

Many authors identified a need to embed the patient within the team to allow for co-design of goals and interventions, which should enhance communication and coordination of the rehabilitation team.^1,4–13,15–17,24–29,31^

#### Motivation – Reflective and automatic

Researchers identified that preparation and training, implementation of standardized goal-setting approaches, adequate time, and inclusive teams can increase goal-setting confidence in patients and providers.^1–17,23–31^ These changes would help shift traditional attitudes and perceptions in support of patient-centred goal-setting. This shift would enhance therapeutic relationships and further promote the use of this practice to help embed it into routine clinical processes. Rewards and incentives to encourage patient and provider participation were indicated in several studies.^1–3,6,7^

A summary of the most prominent barriers and facilitators, organized by the Capability, Opportunity, Motivation Behaviour Model, and level (patient, provider, organizational), is presented in Table 2.

**Table 2.** Summary of level barriers and facilitators to patient-centred goal-setting at the at the patient, healthcare provider and organizational level.

Patient level barriers and facilitators			
COM-B sub-component	Barrier frequency*	Barriers	Facilitators
**Capability –** **physical**	✓✓	Lack of training^1,3–5,8,13,15^	Training/resources^1,3–5,8,13,15^
**Capability –** **psychological**	✓✓	Lack of knowledge^1,3–5,8,13,15^	Education^1,3–5,8,13,15^
	✓✓✓	Patient's role unclear^1,4,6,8,9,13,15,16,25^	Clarify patient role^1–7,9–11,13–17,24^
**Opportunity –** **physical**	✓✓	Patient not part of clinical team^4,5,7,15–17,27,30,31^	Embed patient in team^4,5,7,15–17,27,30,31^
	✓✓	Limited time and access to provider^1–3,11,29^	Not identified
	✓✓	Technical language^1–8,10,11,14–17,26,27,29–31^	Simplify/explain language^1–8,10,11,14–17,26,27,29–31^
**Opportunity –** **social**	✓✓	Goal-setting not optimizing therapeutic relationships^1–7,9–11,13–17,24^	Not identified
**Motivation –** **reflective**	✓✓✓	Different perception of goal-setting than provider^1–3,5–9,11,14,16,17^)	Patients prepared for participation^1–4,9–11,14,25–28,31^
	✓✓✓	Expect “expert” provider, undervalue own experience^1–7,9–11,13–17,24^	
**Motivation –** **automatic**	✓✓	Afraid to share hopes, challenges^3,4,7,13,27–29,31^	Not identified
**HCP barriers and facilitators**			
**COM-B sub-component**	**Barrier** frequency*	**Barriers**	**Facilitators**
**Capability –** **physical**	✓✓✓	Lack of provider training and skills^1–3,5–7,9–17,26,29,31^, no standard process^5,6,8,13,14,16,23^	Training/resources, standard, evidence-informed guideline^1–6,10,11,13–15,17,27,29,30^
**Capability – psychological**	✓✓✓	Lack of provider knowledge: goal-setting, behaviour change, theory^1,3,4,6–8,10,11,14,15,25,27,28^	Education^1,3,4,6–8,10,11,14,15,25,27,28^
**Opportunity – physical**	✓✓	Clinical documentation^1–4,6,8,10,11,14,26,29^	Unspecified
	✓✓	Goals not used to enhance interprofessional team functioning^1,5,6,9–11,21,27,30^	Inclusive team structures, processes^1,4–13,15–17,24–29,31^
	✓✓	Limited time, workload pressures^1–3,5,6,8,9,13,24,28^	Redesign workflow^1,3,6–8,15,24,27,29^
**Opportunity –** **social**	✓✓	Goals not optimizing therapeutic relationship^1,3,8,11,12,16,23,31^	See reflective motivation
**Motivation –** **reflective**	✓✓ ✓✓✓ ✓✓✓	HCPs believe: They are patient-centred^1–3,10,12,14,15,31^ They are the “expert” and must manage patient expectations ^1–5,7–12,14–16^ Patients' incapable of participating ^1–4,8–10,12,14,16,17,26,29–31^	Clinical guideline, templates, tools, provider training^1–6,10,11,13–15,17,27,29,30^
	✓✓	Technical language^1–8,10,11,14–17,26,27,29–31^	Simplify/explain language^1–8,10,11,14–17,26,27,29–31^
**Motivation –** **automatic**	✓✓✓	Lack of confidence^1,3,6–10,12–17,27–31^	Measure/recognize provider performance^1–3,6,7^
**Organizational barrers and facilitators**			
**COM-B sub-component**	**Barrier** frequency*	**Barriers**	**Facilitators**
**Capability –** **physical**	✓✓✓	No standardized approach^4,5,7,12,13,16,17,23,24,26,29,31^. Patients not prepared to participate^1,2,5,6,12,14^	Standardized guideline^4,5,12,13,16,17,23,24,26^. Patient education^1,2,5,6,12,14^
**Capability –** **psychological**	✓✓	Theory not effectively integrated into education and practice^1–6,10,11,13–15,17,27,29,30^	Guideline, evidence informed, theory driven education^1–6,10,11,13–15,17,27,29,30^
**Opportunity –** **physical**	✓✓✓	Lack of provider time^1–3,8,10–12,14,15,17,24,29,31^ and high-productivity targets^1,3,4,6–8,15,24,27,28^	Adequate time^1,3–8,15,24,27,29^, productivity tracking and clinical routine adaption^1,3,6–8,15,24,27,29^
	✓✓	Clinical documentation^3,6–8,12,16,29^	Clinical documentation/ tools^1,6,12,15,16,29,31^.
**Opportunity –** **social**	✓	Impact of patient culture on goal-setting not understood^2^	Cultural impact on goal-setting requires investigation^2^
**Motivation - reflective**	✓	Standard organizational goal-setting approach absent but would enable performance measurement and recognition^3^	
**Motivation –** **automatic**	✓	Impact of patient culture on goal-setting requires investigation^2^	

*✓✓✓ Theme present in > 12 studies; ✓✓ theme present in 6–12 studies; ✓ theme present in 1–5 studies.

COM-B: Capability, Opportunity, Motivation Behaviour Model; HCP: healthcare provider.

## Discussion

In this scoping review we found barriers and facilitators to patient-centred goal-setting at the patient, provider, and organizational level. Patient barriers relate to capability to understand one's condition, goal-setting and rehabilitation^1–4,8,13,15^ and lack of opportunities within the clinical context to participate.^7,15–17,27,30,31^ Barrier themes for providers include the belief that they provide patient-centred care and engage patients in goal-setting,^1–4,8–15,26,29,31^ lack of facilitation skills,^1–4,6,7,9–17,26,29,31^ and limited integration of goal-setting into clinical routines.^1,3,8,11,12,16,23,31^ Organizational barriers include a lack of clinical goal-setting guidelines,^1,2,5–8,12–15,23,24,26,29^ insufficient patient preparation,^1–4,8,13,15^ and limited time for goal-setting^1–3,8,10–12,14,15,17,24,29,31^ due to high productivity expectations.^1,3,4,6–8,15,24,27,28^ Facilitators included clinical goal-setting guidelines,^1,3–7,12,13,16,17,23,24,26^ training and education in goal-setting for providers^1–6,10,11,13–15,17,27,29,30^ and patients^1,3–5,8,13,15^, revised clinical routines,^1,3,6–8,15,24,27,29^ performance monitoring,^1–3,6,7^ adequate time,^1–3,8,10–12,14,15,17,24,29,31^ and resources.^1–3,11,29^

The review findings suggest a focus on developing interventions directed toward provider behaviours to improve application of patient-centred goal-setting. A breadth and depth of research about barriers and facilitators to patient-centred goal-setting on which to design behaviour change interventions was identified,^1–17,23–31^ thus, continued study of barriers and facilitators is not likely to add novel information to the literature. In particular, findings about reflexive motivation support the primary area for intervention should be providers’ attitudes and beliefs about goal-setting.^1–4,8–10,12,14,16,17,26,29–31^ Providers’ motivation was further influenced by capability barriers (skills)^1–6,10,11,13–15,17,27,29,30^ and opportunity areas (time, team processes).^1–3,8,10–12,14,15,17,24,29,31^ Lloyd et al.^10^ reported patient-centred goal-setting can contribute to a positive patient experience but is highly dependent on the provider's experience, skills, and attitude.

Many of the patient barriers identified in the review are modifiable through provider practice.^3–7,24^ Patients are inclined to undervalue their ability to gain the appropriate knowledge to fully participate, and providers underrate patients’ complementary expertise and life context.^1–4,8,13,15^ Providers can enhance patient capability to set goals by educating them about their condition and the goal-setting process and using goal-setting tools to support.^1–4,8,13,15–17^ These efforts would make the patient contribution visible, and balance power differentials in the clinical encounter.^1–3,6,7,9,10,15^

Providers believe they deliver patient-centred goal-setting and express a desire for patients to be more involved, but in practice patients are not actively engaged.^1–4,6,10,11,13–15,17^ The providers know what patient-centred goal-setting entails, however evidence demonstrates that positive motivations have not translated into practice wherein patient-centred goals are foundational to service delivery.^1,3,7,9–11^ Clinical goal-setting guidelines, integrated into local clinical routines, could concretely support a necessary shift in individual behaviours and change traditional team culture.^1–17,23,24,26,29,31^

Our review suggests that providers require enhanced clinical skills to improve their ability to deliver patient-centred goal-setting.^1–4,6,7,9–17,26,29,31^ This includes competency in goal-setting facilitation tailored to patient's needs and clinical context. This approach indicates that patient participation is an expectation.^1,3–5,8,13,15,23,31^ Goal-setting intervention training has been shown to improve provider knowledge, attitudes, and confidence in collaborative goal-setting.^5–7,13,16,17,23,24,26,29,31^ However, practice change is often not sustained^32,33^ without frequent follow-up, including audit and feedback.^32,33^ Methods for improving competency through continued support require more research.

The findings of this review also suggest organizations and implementation leadership are important to enable patient-centred goal-setting.^1–3,5–7,29^ This can include: clinical goal-setting guideline implementation^1,2,5–8,12–15,23,24,26,29^; time to complete goal-setting^1–3,8,10–12,14,15,17,24,29,31^ management of workload expectations^1,3,4,6–8,15,24,27,28^; clinical resources to support goal-setting^1–3,11,29^; enhanced clinical documentation^1,6,12,15,16,29,31^ to utilize patient goals to coordinate the team; and provider performance monitoring and recognition. Organizations have a role in preparing patients to fully participate in goal-setting, shifting current biomedical care models, and normalizing goal-setting.^1–3,5–7^

Since this scoping review only included review studies, it is possible the detailed findings of an individual study were not included. The convergent findings across reviews provide confidence our results reflect the common patterns in the literature. These findings would benefit from understanding in diverse contexts (hospitals, community) before they are applied in specific rehabilitation settings. Most findings are drawn from evidence in the adult stroke population and traditional rehabilitation professionals, thus the findings may be less applicable as a target for intervention to other contexts, such as paediatrics and with emerging professions such as recreational therapy or child life.

We identified a few knowledge gaps in the research including, identification of optimal team structures for effective goal-setting; the impact and role of caregivers (paid and unpaid); and understanding the impact of patient-centred goal-setting with culturally diverse clients.

In this scoping review we used a multi-level, theoretically informed approach to synthesize the evidence about barriers and facilitators to patient-centred goal-setting in rehabilitation. This review is the first step toward developing theory-based and evidence-based interventions to improve the application of patient-centred goal-setting in clinical rehabilitation. We identified that providers should be the priority group for intervention and, their attitudes and beliefs about goal-setting coupled with practice competency were the most prominent barriers to practice. Patient barriers are modifiable through provider support, enabling capability-building and creation of clinical opportunities for goal-setting participation. Further, leaders and organizations are key in creating optimal clinical goal-setting environments.

**Clinical messages**

- Clinicians have positive beliefs about patient-centred goal-setting but find it difficult to implement in daily practice.
- Organizations create the implementation environment to address clinician barriers, such as ongoing coaching to improve competence, changes to clinical routines to integrate the practice and, individual and team performance management and rewards.
- Patients need supports to learn what goals are and how to express them prior to clinical encounters.

## Supplemental Material

sj-docx-1-cre-10.1177_02692155221121006 - Supplemental material for Facilitators and barriers to patient-centred goal-setting in rehabilitation: A scoping review Click here for additional data file. Supplemental material, sj-docx-1-cre-10.1177_02692155221121006 for Facilitators and barriers to patient-centred goal-setting in rehabilitation: A scoping review by L. Crawford, J. Maxwell, H. Colquhoun, S. Kingsnorth, D. Fehlings, S. Zarshenas, S. McFarland and Nora Fayed in Clinical Rehabilitation

sj-docx-2-cre-10.1177_02692155221121006 - Supplemental material for Facilitators and barriers to patient-centred goal-setting in rehabilitation: A scoping review Click here for additional data file. Supplemental material, sj-docx-2-cre-10.1177_02692155221121006 for Facilitators and barriers to patient-centred goal-setting in rehabilitation: A scoping review by L. Crawford, J. Maxwell, H. Colquhoun, S. Kingsnorth, D. Fehlings, S. Zarshenas, S. McFarland and Nora Fayed in Clinical Rehabilitation

sj-docx-3-cre-10.1177_02692155221121006 - Supplemental material for Facilitators and barriers to patient-centred goal-setting in rehabilitation: A scoping review Click here for additional data file. Supplemental material, sj-docx-3-cre-10.1177_02692155221121006 for Facilitators and barriers to patient-centred goal-setting in rehabilitation: A scoping review by L. Crawford, J. Maxwell, H. Colquhoun, S. Kingsnorth, D. Fehlings, S. Zarshenas, S. McFarland and Nora Fayed in Clinical Rehabilitation

sj-docx-4-cre-10.1177_02692155221121006 - Supplemental material for Facilitators and barriers to patient-centred goal-setting in rehabilitation: A scoping review Click here for additional data file. Supplemental material, sj-docx-4-cre-10.1177_02692155221121006 for Facilitators and barriers to patient-centred goal-setting in rehabilitation: A scoping review by L. Crawford, J. Maxwell, H. Colquhoun, S. Kingsnorth, D. Fehlings, S. Zarshenas, S. McFarland and Nora Fayed in Clinical Rehabilitation
