# Supplementary material for: Facilitators and barriers to patient-centred goal-setting in rehabilitation: A scoping review
Source: Clin Rehabil. 2022 Aug 25;36(12):1694–704. doi: 10.1177/02692155221121006 (PMC9574028; doi:10.1177/02692155221121006)
Supplement: sj-docx-1-cre-10.1177_02692155221121006 - Supplemental material for Facilitators and barriers to patient-centred goal-setting in rehabilitation: A scoping review [file sj-docx-1-cre-10.1177_02692155221121006.docx]

**Appendix D Summary of Included Studies Organized by Study Aim**

| **Primary Studies: Barriers and facilitators to patient-centred goal-setting (4)** | | | | |
| --- | --- | --- | --- | --- |
| **Author/Year/Country** | **Review Type** | **Participants (n)** | **Primary studies (n) Study type** | **Study Aims Setting** |
| **Plant et al.**  **2016**  **UK** | Systematic review and meta-synthesis | Patients (88)  Caregivers (25)  Provider (89) | Qualitative (9) | Barriers, facilitators to goal-setting in stroke, ABI rehabilitation |
| **Rosewilliam et al.**  **2011**  **UK** | Systematic review and synthesis | Patients (983)  Provider (335)  Caregivers (25) | Qualitative (18)  Quantitative (8)  Mixed methods (1) | Application, barriers, facilitators to patient-centred goal-setting in stroke rehabilitation. |
| **Sugavanam et al.**  **2013**  **UK** | Systematic review | Patients (614)  Provider (43)  Caregivers (38) | Quantitative (11)  Qualitative (6) | Effectiveness, barriers, facilitators to goal-setting in stroke rehabilitation |
| **Secondary Sources A: Experience and effectiveness (8)** | | | | |
| **Author/Year/**  **Country** | **Review Type** | **Participants (n)** | **Primary studies (n) Study type** | **Study Aims Setting** |
| **MacDonald et al.**  **2013**  **New Zealand** | Literature review | Patients (176)  Provider (49)  Caregivers (3) | Qualitative (14)  Mixed methods (3) | Barriers, facilitators to patient engagement in stroke rehabilitation. |
| **Forgea et al.**  **2021**  **USA** | Integrative  review | Adult stroke | Qualitative (25) | Barriers and facilitators to engagement in rehabilitation among stroke survivors.  Inpatient and rehabilitation setting |
| **Lloyd et al.**  **2018**  **UK** | Systematic review and meta-synthesis | Adult patients (24)  Family (7)  Provider (28) | Qualitative (4) | Goal-setting experiences in stroke rehabilitation. |
| **Maribo et al.**  **2020**  **Denmark** | Systematic review | Patients (46)  Provider (7) | Qualitative (4) | Goal-setting experiences in SCI rehabilitation. |
| **Smit et al.**  **2019**  **Netherlands** | Systematic review and meta-analysis | Geriatric patients (1915) | Quantitative (14)  RCTS (10) | Goal-setting impact on physical functioning, quality of life, duration in patients with acquired disability. |
| **Rose, et al.**  **2017**  **UK** | Systematic review | Adult patients (786)  Family (7)  Provider (120) | Qualitative (8)  Quantitative (6)  Mixed methods (1) | Application, experience of shared decision-making in rehabilitation goalsetting. |
| **Levack et al. 2006**  **New Zealand** | Systematic review | Adult patients (1017) | RCTs (19) | Effectiveness of goal panning in rehabilitation with adults with acquired disability - hospital, community. |
| **Levack et al.**  **2006**  **New Zealand** | Systematic Review | Adult Patients (2005) | Quantitative and Qualitative (19) | Effectiveness of goal-setting outcomes in adults with acquired disability hospital and community settings |
| **Levack et al. 2015**  **New Zealand** | Systematic review | Adults (2846) | RCTs (27) | Goal-setting strategies, outcomes in rehabilitation for acquired disability. |
| **Secondary Sources B: Approaches to patient-centred goal-setting and associated tools (10)** | | | | |
| **Author/Year/Country** | **Review Type** | **Participants (n)** | **Primary studies (n) Study type** | **Study Aims Setting** |
| **Nguyen et al.**  **2019**  **Canada** | Rapid review of literature. | Children/youth  Caregivers  Provider (n/a) | Qualitative (13)  Quantitative (2)  Mixed methods (1) | Use of ICF framework in goalsetting in pediatric rehabilitation. |
| **Kamioka et al.**  **2009**  **Japan** | Systematic Review | Adult patients  Provider  (N not specified) | Qualitative and quantitative (165) | Goal-setting methods, tools (GAS, COPM) in physical therapy in stroke rehabilitation. |
| **Tang et al.**  **2014**  **Australia** | Mixed methods systematic review | Adult Caregivers (3183) | Quantitative (15)  Qualitative (2) | Goal-setting, outcomes with caregivers in community rehabilitation. |
| **Evans**  **2012**  **UK** | Literature review | Patients  Provider  (N not specified) | Qualitative and quantitative (8) | Goal-setting in ABI rehabilitation. |
| **Hodgetts et al.**  **2017**  **Canada** | Scoping review and chart review | Children (83) | Qualitative & quantitative (7)  Other sources (4) | Strategies, tools in goal-setting for Autism. |
| **Hurn et al.**  **2006**  **UK** | Systematic review | Adult patients (1351) | Quantitative studies (15) | Evaluate goalsetting as an outcome measure in adults - physical and neurological rehabilitation in hospital, community. |
| **Constand et al.**  **2014**  **Canada** | Scoping review | Provider (N/A) | Qualitative (11)  Quantitative (6)  Mixed methods (1) | Integration of ICF into goal-setting practices, outcomes in rehabilitation |
| **Pritchard-Wiart et al.**  **2018**  **Canada** | Scoping review | Children  Family/Caregiver  Provider (N/A) | Quantitative (35)  Qualitative (8)  Mixed methods (6)  Reviews (13) | Theory use, evaluation of goalsetting processes, outcomes in pediatric rehabilitation. |
| **Stevens et al. 2013**  **Netherlands** | Systematic review | Adult patients (469)  Provider (145) | Qualitative (11) | Patient measurement instruments in goalsetting, impact on provider practice – physical, neurological rehabilitation |
| **Prescott et al.**  **2015**  **Australia** | Systematic scoping review | Adults (67)  Family (10)  Provider (54) | Qualitative (62)  Quantitative (24) | Identify, evaluate goal- setting approaches in ABI rehab. |
| **Secondary Sources C: Theory and goal-setting in rehabilitation (4)** | | | | |
| **Author/Year/Country** | **Review Type** | **Participants (n)** | **Primary studies (n) Study type** | **Study Aims & Setting** |
| **PritchardWiart et al.**  **2019**  **Canada** | Literature review | Unknown (N/A) | Unknown (N/A) | Relevant goalsetting theories, implications for pediatric rehabilitation |
| **Siegert et al.**  **2004**  **New Zealand** | Literature review | Unknown (N/A) | Self-regulation theory (N/A) | Goal-setting + self-regulation theory,  its use in rehabilitation. |
| **Scobbie et al.**  **2009**  **UK** | Literature review | Unknown (N/A) | Quantitative  (24) | Five behaviour change goal-setting theories reviewed in chronic conditions/ABI rehabilitation |
| **Siegert et al.**  **2004**  **New Zealand** | Literature review | Unknown (N/A) | Social cognition theory (N/A) | Review of rehabilitation goalsetting theories. |

*Notes:* Healthcare provider (Provider), Acquired Brain Injury (ABI), Goal Attainment Scaling (GAS),

International Classification of Functioning (ICF); Spinal Cord Injury (SCI), (N/A) Not Specified
