# Supplementary material for: Facilitators and barriers to patient-centred goal-setting in rehabilitation: A scoping review
Source: Clin Rehabil. 2022 Aug 25;36(12):1694–704. doi: 10.1177/02692155221121006 (PMC9574028; doi:10.1177/02692155221121006)
Supplement: sj-docx-3-cre-10.1177_02692155221121006 - Supplemental material for Facilitators and barriers to patient-centred goal-setting in rehabilitation: A scoping review [file sj-docx-3-cre-10.1177_02692155221121006.docx]

**Supplementary File 2. Scoping Review Search Strategy**

**MEDLINE**

[Mesh Headings] (Goals OR Patient Participation OR Keyterms [kf,tw] (Goal settings OR plans OR negotiat* OR discuss* OR propos* OR prescrib* OR develop* OR formulat* OR establish* OR identif* OR action plan* OR coping plan* OR shared decision mak*)) AND [Mesh Headings] (Stroke rehabilitation OR Rehabilitation / organization & administration OR Keyterms [kf,tw] Rehabilitation) Filter applied for systematic reviews, reviews, qualitative studies as publication types. Results: 506

**EMBASE**

[Emtree] (hospital planning OR planning OR health care delivery OR patient care planning OR Keyterms [kf,tw] (Goal settings OR plans OR negotiat* OR discuss* OR propos* OR prescrib* OR develop* OR formulat* OR establish* OR identif* OR action plan* OR coping plan* OR shared decision mak*)) AND [Mesh Headings] (Stroke rehabilitation OR Rehabilitation / organization & administration OR Keyterms [kf,tw] Rehabilitation) Filter applied for systematic reviews, reviews, qualitative studies as publication types Results: 870

**CINAHL**

[Cinahl subject headings] (Goal setting OR teamwork OR collaboration OR patient centred care OR professional-patient relations OR Keyterms [kf,tw] (Goal settings OR plans OR negotiat* OR discuss* OR propos* OR prescrib* OR develop* OR formulat* OR establish* OR identif* OR action plan* OR coping plan* OR shared decision mak*)) AND [Cinahl subject headings] Rehabilitation) Filter applied for systematic reviews, reviews, qualitative studies as publication types Results: 193

**PSYCHINFO**

[PsychInfo subject headings] (Goal setting OR Collaboration OR health care services OR client centered therapy OR patient centred care OR Keyterms [Id, ti, ab] (Goal settings OR plans OR negotiat* OR discuss* OR propos* OR prescrib* OR develop* OR formulat* OR establish* OR identif* OR action plan* OR coping plan* OR shared decision mak*)) AND [PsychInfo subject headings] Rehabilitation) Filter applied for systematic reviews, reviews, qualitative studies as publication types Results: 113

**COCHRANE Database**

| #1 | MeSH descriptor: [Goals] explode all trees | MeSH | 741 |
| --- | --- | --- | --- |
| #2 | MeSH descriptor: [Organizational Objectives] explode all trees | MeSH | 48 |
| #3 | MeSH descriptor:  [Organizational Objectives] explode all trees | MeSH | 48 |
| #4 | MeSH descriptor: [Patient Participation] explode all trees | MeSH | 1450 |
| #5 | MeSH descriptor: [Hospital Planning] explode all trees | MeSH | 1 |
| #6 | MeSH descriptor: [Patient Care Planning] explode all trees | MeSH | 1741 |
| #7 | goal*:ti,ab | Limits | 39685 |
| #8 | (goal-setting*):ti,ab | Limits | 2148 |
| #9 | #1 OR #2 OR #3 OR #4 OR #5 OR #6 OR #7 OR #8 | Limits | 42638 |
| #10 | MeSH descriptor: [Rehabilitation] explode all trees | MeSH | 38197 |
| #11 | REHABILITAT*:TI,AB | Limits | 37112 |
| #12 | #10 OR #11 | Limits | 68878 |
| #13 | #9 AND #12 | Limits | 3818 |
| **Results: 8** |  |  |  |
