# Supplementary material for: Facilitators and barriers to patient-centred goal-setting in rehabilitation: A scoping review
Source: Clin Rehabil. 2022 Aug 25;36(12):1694–704. doi: 10.1177/02692155221121006 (PMC9574028; doi:10.1177/02692155221121006)
Supplement: sj-docx-4-cre-10.1177_02692155221121006 - Supplemental material for Facilitators and barriers to patient-centred goal-setting in rehabilitation: A scoping review [file sj-docx-4-cre-10.1177_02692155221121006.docx]

**Supplementary File 3. PRISMA Flowchart of the Systematic Search and Screening Process**

Articles identified through database searching

Medline: 506

CINHAL:193

Embase: 870

PsychINFO:113

Cochrane: 8

(n= 1691)

Studies included in literature review

(n = 26)

**Screening**

1137 articles screened title and abstract assessed

1092 articles excluded

Full-text articles accessed for eligibility

(n =44)

Full-text articles excluded with reasons: (n=18)

- Abstract English but full-text in German (n=1)
- Conference presentations proceedings (n=2)
- Commentary (n=1)
- Goalsetting not the phenomenon of interest (n=10)
- Specific review of Goal Management Training method (n=4)

**Eligibility**

1693 articles imported into EndNote

(556 duplicated discarded)

(n=1137)

**Included**

**Identification**

Additional articles identified through other resources

(n=2)
